# Pathological Reporting of Radical Prostatectomy Specimens Following ICCR Recommendation: Impact of Electronic Reporting Tool Implementation on Quality and Interdisciplinary Communication in a Large University Hospital

**DOI:** 10.3390/curroncol29100571

**Published:** 2022-09-30

**Authors:** Caroline Richter, Eva Mezger, Peter J. Schüffler, Wieland Sommer, Federico Fusco, Katharina Hauner, Sebastian C. Schmid, Jürgen E. Gschwend, Wilko Weichert, Kristina Schwamborn, Dominik Pförringer, Anna Melissa Schlitter

**Affiliations:** 1 Institute of General and Surgical Pathology, Technische Universität München, Trogerstr. 18, 81675 Munich, Germany; 2 Smart Reporting GmbH, 80538 Munich, Germany; 3 Department of Radiology, LMU University Hospital, 81377 Munich, Germany; 4 Department of Urology, Klinikum Rechts der Isar, Technische Universität München, 81675 Munich, Germany; 5 Clinic and Policlinic for Trauma Surgery, Klinikum Rechts der Isar, Technische Universität München, Ismaninger Straße 22, 81675 Munich, Germany

**Keywords:** prostate cancer, pathological reporting, structured reporting templates, quality improvement

## Abstract

Prostate cancer represents one of the most common malignant tumors in male patients in Germany. The pathological reporting of radical prostatectomy specimens following a structured process constitutes an excellent prototype for the introduction of software-based standardized structured reporting in pathology. This can lead to reports of higher quality and could create a fundamental improvement for future AI applications. A software-based reporting template was used to generate standardized structured pathological reports of radical prostatectomy specimens of patients treated at the University Hospital Klinikum rechts der Isar of Technische Universität München, Germany. Narrative reports (NR) and standardized structured reports (SSR) were analyzed with regard to completeness, and clinicians’ satisfaction with each report type was evaluated. SSR show considerably higher completeness than NR. A total of 10 categories out of 32 were significantly more complete in SSR than in NR (*p* < 0.05). Clinicians awarded overall high scores in NR and SSR reports. One rater acknowledged a significantly higher level of clarity and time saving when comparing SSR to NR. Our findings highlight that the standardized structured reporting of radical prostatectomy specimens, qualifying as level 5 reports, significantly increases objectively measured content quality and the level of completeness. The implementation of nationwide SSR in Germany, particularly in oncologic pathology, can serve pathologists, clinicians, and patients.

## 1. Introduction

Prostate cancer is one of the most common malignant tumors and accounts for 25% of all new cancer cases in male patients in Germany [1]. Pathological examination and reporting following radical prostatectomy play a major role in case management. Excellent evidence-based templates for radical prostatectomy specimens have been published by interdisciplinary experts of leading professional societies, including the International Collaboration on Cancer Reporting (ICCR) [2,3,4], and the advantages of introducing a standardized reporting in pathology have been highlighted for different tumor types in several studies [5,6,7,8,9,10,11]. As shown in a recently published study by Fronhoffs et al., a uniform reporting structure and the consistent diction of findings are of great importance for clinicians and aid in communication between pathologists and clinicians [12]. A recently published dataset from the Netherlands demonstrates that the use of standardized structured reporting results in improved patient care and prognosis of patients with colorectal cancer [13]. Given the increasing importance of machine learning and artificial intelligence (AI) in diagnostics, oncology [14], and medicine in general, the establishment of fully structured reports using electronic tools yielding large datasets is of particular interest [15,16]. The clinical relevance of AI is highlighted by the first ever United States Food and Drug Administration (FDA) approval for an AI-based pathology tool to detect prostate cancer received by Paige Inc. in 2021 [17]. Despite the obvious advantages in patient care, interdisciplinary communication, high importance for precision medicine [18], and structured data as the first step towards digitized pathology, most pathological reports are currently still written as narrative free-text reports (narrative reports/NR), thus reflecting the individual structure and diction of the pathologist in charge.

Annually, more than 400 radical prostatectomy specimens reach the Institute of Pathology at Technische Universität München. About 20 residents and senior pathologists generate different versions of reports on radical prostatectomy specimens. The lack of standardized structure yields discrepancies between reports of both inter- and intrapersonal nature. Prostate cancer surgery and radical prostatectomy follow a structured process [19] and therefore constitute an excellent prototype for a pilot study of the introduction of software-based standardized structured reporting (SSR) in pathology. 

In this single-center study, we aim to investigate the impact of introducing an electronic reporting tool on overall reporting quality and the clinicians’ perception by comparing traditional narrative and innovative standardized structured pathology reports. In addition, the potential use of generated datasets for future AI applications is discussed.

## 2. Materials and Methods

The study was approved by the ethics committee of the TUM school of medicine, Technische Universität München, Munich, Germany (approval number 2022-176-S-NP). 

### 2.1. Study Design and Patients

This pilot study is based on a combined retrospective and prospective analysis on histopathological reports of patients treated via radical prostatectomy at the University Hospital Klinikum rechts der Isar of the Technische Universität München (TUM), Germany. 

The first retrospective cohort contained 101 consecutive patients treated between January and March 2019. Histopathological reports were generated at the Institute of Pathology at TUM by 12 individual board-certified pathologists. All reports were composed in a narrative style (referred to as “narrative reports/NR”) in accordance to the WHO Classification of Tumours of the Urinary System and Male Genital Organs (4th Edition, 2016) [20] without a predefined structure.

The second prospective cohort consisted of randomly selected patients treated between February and July 2021 (*n* = 33) whose pathology reports were created by a single board- certified pathologist (AMS) using a software-based structured reporting tool based on the recently published ICCR template for radical prostatectomy specimens [21] (therefore referred to as “standardized structured reports/SSR”).

Patients with neoadjuvant therapy (*n* = 2) and one patient without histological evidence for malignant tumor in the specimen were excluded from the study leading to a total number of *n* = 131.

### 2.2. Structured Reporting Software

**Figure 1:**
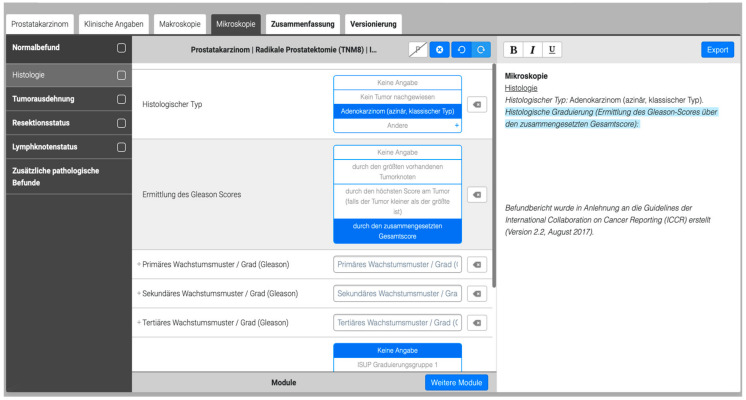
Screenshot of the digital template for pathological reporting of radical prostatectomy specimen based on the Smart Reporting software (original template, German, Smart Reporting GmbH, Munich, Germany, version 1). The black box on the left side shows the overall structure of the template (e.g., histology, tumor size, resection margin). An exemplary drop-down menu (histological findings) is shown in the middle, and the automatically created text file is shown at the right side. In addition, all data entries are stored in a database (not shown).

For this study, a software-based reporting template for prostatectomy specimens following ICCR recommendations [21] including drop-down menus, predefined information fields, and boxes was provided by Smart Reporting GmbH [22] as shown in Figure 1.

### 2.3. Analysis of Overall Content Quality

To investigate the content quality of the individual reports, all elements were evaluated based on the recently published, internationally validated and evidence-based pathology dataset of the ICCR for cancer reporting of radical prostatectomy specimens [21].

In detail, a total of 23 required and recommended categories and 9 subcategories (17 considered as “required” and 15 as “recommended” by ICCR) were defined and scored in all NR and SSR (*n* = 131). Due to different pathological tumor stages, scores between 23 and 32 points were possible. The individual reports were evaluated by awarding 0 (not addressed at all), 0.5 (answered incompletely or indirectly), or 1 point (answered explicitly and completely) for each category resulting in a total quality score for every report expressed in percentage of the maximum score.

### 2.4. Analysis of Completeness of Categories

The different report (sub-) categories were assessed individually by comparing NR and SSR on a feature level. One point was awarded only if the required information was clearly stated (complete as well as explicit). Otherwise, this category yielded 0 points. Each category received a total score, expressed as percentage for better comparability.

The diagnostic quality of the reports was not addressed in the current study.

### 2.5. Analysis of Report Perception by Clinicians

To investigate clinician’s perception, 62 reports (all 32 SSR and 30 NR reports, randomly selected from the NR cohort) were evaluated by two board-certified urologists using a questionnaire (as seen in Appendix A) addressing completeness, time saving, quality, comprehensibility, and clarity. To prevent bias, all patient data, surgeon names, and date of surgery were rendered invisible. In detail, four questions targeting different aspects of the report were addressed in the questionnaire using a scale based on German school grades from 1 (very good/excellent) to 6 (insufficient) and one question was designed with a scale from 1 to 4 (details see Appendix A). Every questionnaire was assigned a total score between five (best score) and 28 (worst score). In all answered questionnaires, three single values were missing. Therefore, the mean value for each question was calculated and imputed for the missing value to avoid a reduction in the sample size.

### 2.6. Statistical Analysis

Statistical analyses were performed using the statistical software Microsoft Excel (Microsoft Corp. Redmond, WA, USA), SPSS (IBM Corp. Armonk, NY, USA), and GraphPad (GraphPad Software Inc. San Diego, CA, USA).

Basic descriptive statistics were used for specification of patients and tumor characteristics. The two-sided Fisher’s exact test was performed to evaluate the significance of NR vs. SSR in each category. Overall mean of NR against SSR was analyzed utilizing a Mann–Whitney *U* test. The two-sided *t*-test was performed to compare individual questionnaire outcomes. *p*-values lower than 0.05 were considered statistically significant.

## 3. Results

134 patients who underwent radical prostatectomy for prostate cancer at the Urology Department, University Hospital Klinikum rechts der Isar of the TUM between 2019 and 2021 were selected. Three patients had to be excluded: two patients received neoadjuvant therapy and one showed no evidence of a malignant tumor in the specimen. Out of the 131 analyzed pathology reports 99 were written in a traditional narrative reporting style (NR *n* = 99) by 12 different board-certified pathologists and 32 reports were prospectively generated by a single board-certified pathologist using a structured reporting software (SSR *n* = 32). Clinicopathological details are given in Table 1. 

### 3.1. Analysis of Overall Content Quality

High total scores of the overall content quality expressed as percentage of the maximum achievable score were observed in both NR and SSR (as seen in Table 2), however, relevant differences can be highlighted. In NR, a mean total content quality score of 73.4% compared to 94.7% in SSR (*p* < 0.0001) was observed. Comparable results were found when exclusively comparing the categories defined as mandatory by the ICCR (*p* < 0.0001).

Furthermore, high variation of wording and terminology was observed in NR compared to SSR due to lack of selection menus with specific terminology provided in the Smart Reporting software.

Some information was considerably harder to find in NR compared to SSR. For example, “Urinary bladder neck invasion” was mentioned indirectly in 21% of NR and missing in 79%, whereas all SSR contained a clear unequivocal statement about this detail. 

Indirectly mentioned means either the information was hard to find due to a different kind of wording and/or position within the text. Both render finding the required information difficult. Additionally information can be overseen or text comprehension can take longer. All together, the findings reflect the character of traditional narrative reporting while non-detectable findings are not always mentioned in the report. Consequently, no definite distinction between a finding that is not detectable by the pathologist and an existing finding accidentally not mentioned in the text can be made.

### 3.2. Analysis of Completeness of Categories

In the next step, completeness of all categories and subcategories was evaluated (as shown in Table 3).

The completeness of the recommended findings “Clinical information” and “Pre-biopsy serum PSA” showed no significant differences between NR and SSR. Whereas “Clinical information” was provided in nearly all reports, PSA values were reported in less than half of the cases. The pathologist has little influence on the presence of the clinical information provided by the clinician.

Macroscopic findings (“Specimen weight”, “Specimen dimensions”, “Seminal vesicles”, and “Lymph nodes”) were complete in all of the narrative and structured reports. However, the above-mentioned categories were provided as a free-text format in NR, and therefore more difficult to find than in the synoptic format of SSR.

Significant differences were observed for distinct microscopic findings, e.g., on the used Gleason-Score (“Indicate how Gleason score is being reported”), the percentage of Gleason pattern 4/5 and presence/absence of lymphovascular invasion (this information was significantly more commonly reported in SSR: *p* < 0.0001, *p* = 0.0144 and *p* < 0.0001, respectively). Moreover, a clear statement on extraprostatic extension, extent (if available), urinary bladder neck invasion and presence/absence of intraductal carcinoma was significantly more common in SSR (details see Table 3).

A few categories were not listed in any of the narrative reports, which can most likely be explained by a difference in standard between international ICCR recommendations and the German Institute of Pathology at the TUM. Six out of the 17 categories considered mandatory (highlighted in bold letters in Table 3) by the ICCR were complete and in the correct format in all NR and SSR, including the clinically most relevant pathological findings “ISUP Grade”, “Margin status” and “Localization of positive margin”,” Lymph node status” and data needed for pathological staging (“Primary tumor” (pT) and “Regional lymph nodes” (pN)). 

### 3.3. Analysis of Report Perception by Clinicians

All SSR (*n* = 32) were compared with a comparable, randomly selected NR sub-cohort (*n* = 30) employing a random number generator. All 62 reports were evaluated by two different board-certified urologists (rater 1 and 2) using a questionnaire addressing completeness, time saving, quality, comprehensibility, and clarity for each individual report. The results of the analysis are shown in Table 4. 

Each report could achieve a total score between 5 being the best and 28 being the worst score. Overall, both NR and SSR received favorable grades by the two raters (mean ranging from 1.03–2.63 per question, total scores ranging from 8.41–10.40). However, relevant differences were observed. SSR received significantly higher total scores by rater 2 compared to NR (8.41 versus 9.93, *p* < 0.0001). In detail, clear differences were observed for time saving aspects (question 2: NR 2.17 versus SSR 1.88, *p* = 0.0021) and clarity of the report (question 5: NR 2.63 versus SSR 1.63, *p* < 0.0001). In contrast, no differences between NR and SSR were observed in the evaluation of first rater’s perception.

## 4. Discussion

Pathological reports serve different purposes: they document pathologists work, inform about diagnoses and staging, serve as interdisciplinary communication tool, and are crucial for clinical management and decision making. Therefore, reports should be well structured, easily understandable, as well as technically correct and complete. However, most pathological reports are currently still written as narrative free-text reports (narrative reports/NR), thus, reflecting the individual reporting style of the pathologist. These narrative reports without defined content and single text fields qualify as level 2 of 6 according to the Ontario scale [15]. The Ontario scale defines six different levels of pathological reports from traditional reports (level 1), to narrative reports with standardized content (e.g., WHO classification; level 2) to formatted reports using a synoptic format (level 3), to more advanced reporting formats using electronic tools/software (level 4), electronic tools in combination with standardized structures datasets (e.g., evidence-based ICCR templates) resulting in discrete data (level 5), and high-end reports combining level 5 requirements with terminology binding (e.g., ICD-0; level 6) [15]. Despite promising data from the widespread use of standardized structure reports (SSR) using electronic reporting tools in the Canadian province Ontario [23,24], the Netherlands [13], and Norway [25,26], SSR is still widely uncommon in Germany. Here, we report the first study of the implementation of a software-based reporting tool of an evidence-based ICCR template for radical prostatectomy specimens in a large University Hospital in Germany qualifying as level 5 reports and evaluate the advantages of SSR to traditional level 2 reports.

The results of our investigation show that traditional narrative reports at our institute are already of high quality and contain most of the information referring physicians need. In detail, we analyzed both the objective content quality and the objective completeness of all NR and SSR reports according to the ICCR guidelines. Significant differences showing the superiority of SSR over NR were highlighted by the overall content quality scores of the evaluated pathology reports. On average, the NR scored 73.43% in quality scores based on information required according to the ICCR, whereas the SSR achieved a mean of 94.76% (*p* < 0.0001) (see Table 2). Likewise, the level of completeness of 10 out of 32 (sub-) categories was significantly higher in SSR (e.g., percentage of Gleason pattern 4/5, presence/absence of lymphovascular invasion; for more details, see Table 3). On the other hand, no differences in the completeness and quality between NR and SSR reports were noticed by two board-certified urologists using a questionnaire to evaluate 30 NR and 32 SSR reports. The discrepancy between the subjective perception of the referring physicians and the objective evaluation of the reports can be explained by the common practice that non-pathological findings or the absence of a pathological finding are generally not mentioned in the report—, e.g., if no lymphovascular invasion is detectable, no statement about the lymphovascular invasion is made. Although this is a widespread practice in the field, this lack of unequivocal data might cause aberration in certification and accreditation processes, and could lead to misunderstanding when an accidentally forgotten information is being considered negative and not being questioned further from the clinical side. This approach appears less serious in categories not influencing therapy decision. However, especially in difficult cases each additional information can impact treatment decision. From a legal perspective, a more complete pathology report is also advantageous. Moreover, lack of information leads to incomplete datasets and less valuable patient cohorts for clinical research.

For referring physicians, it is important to receive a clear and precise statement from pathologists. This was addressed in our questionnaire which was answered by two board-certified urologists. Whereas one of the raters noticed no differences in all five categories addressed in the above-mentioned questionnaire (completeness, time saving, quality, comprehensibility, and clarity), the other rater observed significantly higher clarity and time saving in SSR compared to NR reports. The time saving aspect might be related to the different formatting of non-structured NR and SSR using a synoptic format and reaches beyond the completeness of ICCR categories. 

During the analysis of all reports (*n* = 131), differences were noticed between NR and SSR, the latter revealing the information at first glance. SSR display a synoptic format with bullet points and are therefore quick and easy to review. In NR, there is a considerable variation depending on the author. Some pathologists use a text-only format, others a combination of complete sentences and a synoptic summary of the most important information at the end of the report. The exact location of clinically relevant information differs considerably between SSR and NR as well as between NR from different pathologists. In SSR, all information crucial for the planning of further treatment is clearly arranged at the end of the report. This can save time in extracting the relevant data for the respective clinician. The lack of synoptic formatting of data goes beyond time saving aspects. On top of that, the introduction and further implementation of artificial intelligence in the field of pathology and other fields of medicine depends on both complete and uniformly structured data [15,16]. Due to non-uniform wording, data extraction in level 2 NR reports is time-consuming, complex, and often requires additional support by humans. In contrast, all SSR reports of the prospective cohort qualify as level 5 reports with synoptic format and discrete data fields. They can be directly deposited in a well researchable database for further investigation. Given the large number of radical prostatectomy specimens sent to our University Hospital, smaller hospitals, and pathological institutes in Germany could benefit from evidence-based datasets in terms of easy comparison of their cases with stored data, especially in difficult cases. Moreover, this data can also be added to central databases (e.g., German Centre for Cancer Registry Data) and therefore support an advanced national and international evaluation system serving population-based healthcare management [15,27].

In addition to advantages for clinical studies and translational research, the implementation of a software-based reporting system serves the pathologist during the decision-making process. The software offers a clear structure with text-fields, drop-down menus, and boxes, thus serving as a checklist during the specimen evaluation. This is of particular interest for young residents and can also serve as a self-monitoring tool for experienced pathologists.

Moreover, new versions and updates of reporting guidelines can be easily incorporated into a software-based reporting program and, therefore, made available for all pathologists using the software. Thus, constant report quality in accordance with the latest standards can be maintained. In the long run, it can be assumed that a structured and software-supported method of reporting will save time in everyday medical practice, benefitting both pathologists and urologists. 

## 5. Conclusions

In conclusion, our pilot study on radical prostatectomy specimen reporting shows that SSR significantly increases objectively measured content quality and level of completeness. Given the currently shown correlation between the use of SSR and improved patient care with higher survival rates in patients with colorectal cancer from the Netherlands [13], the implementation of nationwide SSR in Germany—in particular, in oncologic pathology—is of high importance for pathologists, clinicians and, most importantly, for patients. 

### Limitations of the Study

The rating urologists were not given any precise information in advance and no expectations were formulated, to not influence the evaluation process. However, due to different conditions in both groups, limitations may arise when comparing the results of this pilot study. Cohort 1 includes more than three times as many subjects as cohort 2. However, the distribution of pT stages shows no relevant difference between the two groups.

The Influence of the examiner-dependent factor could be greater in the SR group, as only one pathologist was responsible for all findings. In addition, the selection of reports evaluated by the two urologists could cause errors. The urologists worked with all of the SR but only one randomly selected third of NR. Additionally, the number of urologists rating the reports is small, so no general statement can be made but a tendency can be shown. Therefore, larger studies are required to confirm our results.

## Figures and Tables

**Table 1:** Clinicopathological characteristics of prostate cancer patients after radical prostatectomy.

Characteristics	Narrative Reports (NR)	Standardized Structured Reports (SSR)
Time period of surgery	January–March 2019	February–July 2021
Patients included	*n* = 99	*n* = 32
Patients excluded	*n* = 2	*n* = 1
Age in years (median (range))	66 (45–84)	64 (49–79)
pT		
pT2a	3 (3%)	1 (3%)
pT2b	2 (2%)	0 (0%)
pT2c	50 (51%)	19 (59%)
pT3a	26 (26%)	7 (22%)
pT3b	18 (18%)	5 (16%)
pN pN0 pN1		
	83 (84%) 16 (16%)	30 (94%) 2 (6%)

**Table 2:** Overview of mean total quality scores achieved in NR and SSR. Mean percentages were compared by performing a Mann–Whitney *U* test. Reports scoring 100% contained all relevant information recommended by ICCR in an explicit and easy to find way. *p*-values < 0.05 were considered statistically significant and are marked with an asterix.

Content Quality Score	Narrative Reports (NR)	Standardized Structured Reports (SSR)	*p*-Values
	*n* = 99	*n* = 32	
All elements			
Mean	73.43%	94.76%	<0.0001 *
Range	57.81–83.33%	91.67–100%	
Mandatory elements only			
Mean	77.70%	95.78%	<0.0001 *
Range	68.75–84.38%	93.33–100%	

**Table 3:** Summary of all investigated categories and subcategories (17 considered as mandatory, highlighted in bold) in narrative (NR) and standardized structured reports (SSR) of radical prostatectomy specimens according to recommendations by the International Collaboration on Cancer Reporting (ICCR), slightly adapted. The two-sided Fisher’s exact test was performed to compare categories in NR with SSR. *p*-values < 0.05 were considered statistically significant and are marked with an asterix.

	Narrative Reports (NR)	Standardized Structured Reports (SSR)	*p*-Values
	*n* = 99	*n* = 32	
Ontario scale level	2	5	
CLINICAL DATA			
Clinical information	95/99 (96%)	32/32 (100%)	0.5716
Pre-biopsy serum PSA	37/99 (37%)	13/32 (41%)	0.8348
MACROSCOPY			
**Specimen weight**	99/99 (100%)	32/32 (100%)	1
Specimen dimensions	99/99 (100%)	32/32 (100%)	1
**Seminal vesicles**	99/99 (100%)	32/32 (100%)	1
**Lymph nodes**	99/99 (100%)	32/32 (100%)	1
→ Laterality	99/99 (100%)	32/32 (100%)	1
Block identification key	99/99 (100%)	32/32 (100%)	1
MICROSCOPY			
**Histological tumor type**	94/99 (95%)	32/32 (100%)	0.3338
**How Gleason-Score is being reported**	5/99 (5%)	32/32 (100%)	<0.0001 *
**Gleason-Score**			
Percentage Gleason pattern 4/5	97/99 (98%)	32/32 (100%)	1
**ISUP Grade**	77/99 (78%)	31/32 (97%)	0.0144 *
	99/99 (100%)	32/32 (100%)	1
Intraglandular extent	94/99 (95%)	32/32 (100%)	0.3338
**Extraprostatic extension**	67/99 (68%)	32/32 (100%)	<0.0001 *
→ Location(s)	25/41 (61%)	11/12 (92%)	0.0765
→ **Extent**	10/41 (24%)	12/12 (100%)	<0.0001 *
**Seminal vesicle invasion**	92/99 (93%)	32/32 (100%)	0.1935
**Urinary bladder neck invasion**	0/99 (0%)	32/32 (100%)	<0.0001 *
Intraductal carcinoma of prostate	0/99 (0%)	32/32 (100%)	<0.0001 *
Lymphovascular invasion	20/99 (20%)	32/32 (100%)	<0.0001 *
**Margin status**	99/99 (100%)	32/32 (100%)	1
→ **Location of positive margin(s)**	29/29 (100%)	4/4 (100%)	1
→ Type of margin positivity			
→ Extent of margin positivity	17/29 (59%)	3/4 (75%)	1
→ Gleason pattern of tumor present at positive margin	1/29 (4%)	4/4 (100%)	<0.0001 *
**Lymph node status**	1/29 (4%)	4/4 (100%)	<0.0001 *
→ Laterality			
→ Maximum dimension of largest deposit	99/99 (100%)	32/32 (100%)	1
	7/16 (44%)	2/2 (100%)	0.4706
	16/16 (100%)	2/2 (100%)	1
PATHOLOGICAL STAGING			
**Primary tumor (pT)**	99/99 (100%)	32/32 (100%)	1
**Regional lymph nodes (pN)**	99/99 (100%)	32/32 (100%)	1
**Distant metastasis (pM)**	0/99 (0%)	11/32 (34%)	<0.0001 *

**Table 4:** Summary of questionnaire evaluation showing mean scores for all narrative (NR, *n* = 30) and standardized structured reports (SSR, *n* = 32) by the two board-certified urologists (rater 1 and 2). The two-sided *t*-test was performed to compare NR and SSR. *p*-values < 0.05 were considered statistically significant and are marked with an asterix.

Question	Subject	Scale	Report Type	Rater 1		Rater 2	
				mean	*p*-value	mean	*p*-value
1	Completeness	1–6	NR SSR	2.03 2.03	0.9637	1.03 1.06	0.6876
2	Time saving	1–4	NR SSR	2.20 2.19	0.9237	2.17 1.88	0.0021 *
3	Quality	1–6	NR SSR	1.90 1.94	0.7265	2.10 1.94	0.1330
4	Comprehensibility	1–6	NR SSR	2.00 1.94	0.5467	2.00 1.91	0.1934
5	Clarity	1–6	NR SSR	2.27 2.06	0.2584	2.63 1.63	<0.0001 *
Total Score		5–28	NR SSR	10.40 10.16	0.5374	9.93 8.41	<0.0001 *

**Figure A1:**
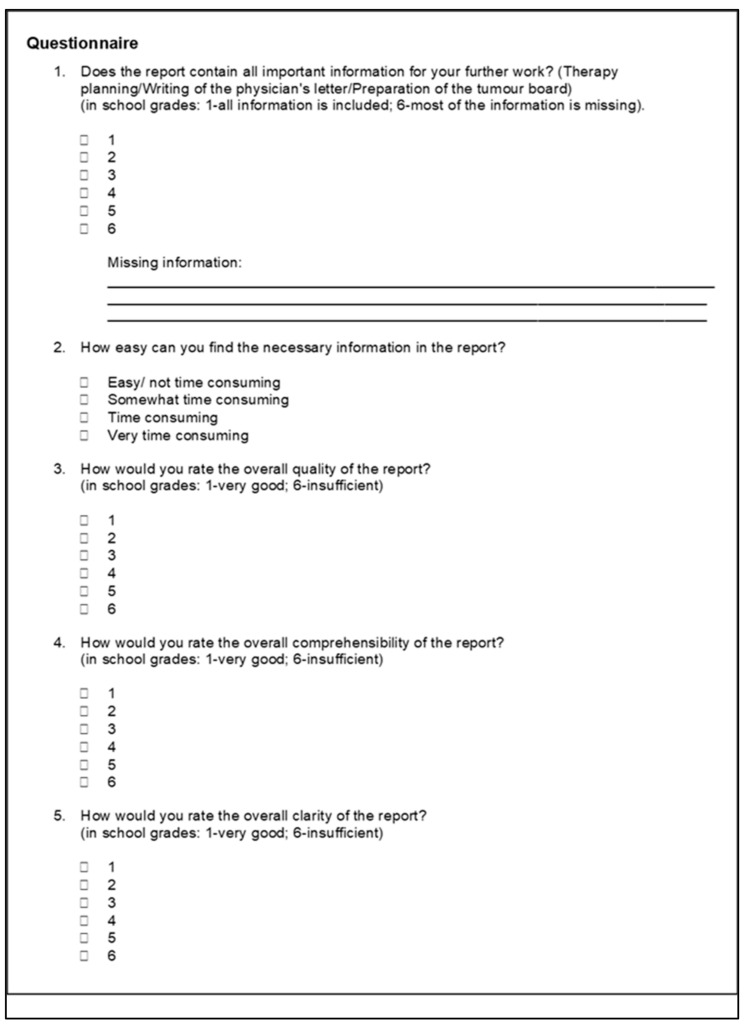
Questionnaire for the evaluation of narrative and structured reports addressing completeness, time saving, quality, comprehensibility, and clarity.

## Data Availability

The data presented in this study are available on request from the corresponding author.
